# A Conantokin Peptide Con-T[M8Q] Inhibits Morphine Dependence with High Potency and Low Side Effects

**DOI:** 10.3390/md19010044

**Published:** 2021-01-19

**Authors:** Zhuguo Liu, Zheng Yu, Shuo Yu, Cui Zhu, Mingxin Dong, Wenxiang Mao, Jie Hu, Mary Prorok, Ruibin Su, Qiuyun Dai

**Affiliations:** 1 Beijing Institute of Biotechnology, Beijing 100071, China; liuzhuguo@126.com (Z.L.); YZYZ.6688@163.com (Z.Y.); o-yys@163.com (S.Y.); zhucuililac@163.com (C.Z.); mxdong64@aliyun.com (M.D.); maomao198655@sina.cn (W.M.); hujie0906@126.com (J.H.); 2 Department of Chemistry and Biochemistry, University of Notre Dame, Notre Dame, IN 46556, USA; mprorok@nd.edu; 3 Beijing Institute of Toxicology and Pharmacology, Beijing 100850, China

**Keywords:** conantokin, con-T[M8Q], NMDA receptor GluN2B subunit, morphine dependence

## Abstract

*N*-methyl-D-aspartate receptor (NMDAR) antagonists have been found to be effective to inhibit morphine dependence. However, the discovery of the selective antagonist for NMDAR GluN2B with low side-effects still remains challenging. In the present study, we report a selective NMDAR GluN2B antagonist con-T[M8Q](a conantokin-T variant) that potently inhibits the naloxone-induced jumping and conditioned place preference of morphine-dependent mice at nmol/kg level, 100-fold higher than ifenprodil, a classical NMDAR NR2B antagonist. Con-T[M8Q] displays no significant impacts on coordinated locomotion function, spontaneous locomotor activity, and spatial memory mice motor function at the dose used. Further molecular mechanism experiments demonstrate that con-T[M8Q] effectively inhibited the transcription and expression levels of signaling molecules related to NMDAR NR2B subunit in hippocampus, including NR2B, p-NR2B, CaMKII-α, CaMKII-β, CaMKIV, pERK, and c-fos. The high efficacy and low side effects of con-T[M8Q] make it a good lead compound for the treatment of opiate dependence and for the reduction of morphine usage.

## 1. Introduction

The *N*-methyl-D-aspartate receptor (NMDAR) is a ligand-gated ion channel composed of two obligatory NR1 subunit and two modulatory NR2(A–D) or NR3(A–B) subunits [1,2]. Its normal activity contributes to numerous processes that facilitate learning and memory, whereas receptor dysfunction is associated with an array of chronic and acute neuropathophysiologies, including ischemic damage, epilepsy, Parkinsonism [3], and opiate addiction [4,5,6]. Increasing evidences demonstrate that NMDAR GluN2B preferentially contributes to pathological processes of glutamaterigic pathway overexcitation compared to GluN2A [5]. Previous studies have also shown that GluN2B is involved in opiate dependence [7,8,9]. Several GluN2B-antagonists, such as ifenprodil [10,11], eliprodil [12], and Ro-256981 [13], have been reported to inhibit morphine dependence in mice or rats with lower side effects than non-subtype selective antagonists. However, current GluN2B antagonists still suffer from relatively high side effects, such as motor deficits and psychiatric disorders [14], which hinder their further applications in clinical practice.

The conantokins, which are derived from *Conus* snail, are a family of naturally occurring gamma-carboxyglutamate (Gla)-rich peptides that specifically antagonize the NMDAR subtypes [15,16]. To this day, twelve conantokins, including con-G, con-T, con-R, and con-L, have been characterized [15,16,17]. We previously reported that con-T, con-G and their variants attenuate the withdrawal syndrome in morphine-dependent mice [18]. However, these conantokins exhibit moderate side effects on mouse motor function even at a low dose (10 nmol/kg) [19], and identifying a potent NMDAR antagonist with low side effects is needed for better clinical application in the treatment of opiate dependence. Con-T[M8Q] (GEγγYQKQLγNLRγAEVKKNA-NH_2_) is a variant of con-T, but has a higher selectivity to GluN1a/GluN2B and GluN1b/GluN2B compared to GluN1a/GluN2A and GluN1b/GluN2A [20], and shows faster dissociation kinetics comparing to con-T or con-G [20]. In addition, con-T[M8Q] efficiently attenuates the expression and development of morphine analgesic tolerance in mice at low doses (5–20 nmol/kg), and it had more potent effects compared with ifenprodil [21].

In the present study, we determined the inhibitory functions of con-T[M8Q] against physical and psychological morphine dependence in vivo using the naloxone-induced jumping and conditioned place preference (CPP) experiments. con-T[M8Q] efficiently attenuates the expression of morphine dependence genes and slows the development of morphine analgesic tolerance in mice at low doses (5–20 nmol/kg), making it dramatically more potent compared to ifenprodil. We also examined and found minimal side effects on coordinated locomotion function, spontaneous locomotor activity and spatial memory.

We also explored the molecular mechanism of con-T[M8Q] inhibition of GluN2B. GluN2B, p-GluN2B, CaMKII-α, CaMKII-β, CaMKIV, nNOS, pERK, and c-fos were previously reported as key signal molecules of morphine dependence related to NMDAR GluN2B [9,22,23] in hippocampus. Using qRT-PCR and Western blot, we found that con-T[M8Q] potently inhibited these signaling molecules at both the transcript and protein level.

Based on our data, we believe that con-T[M8Q] may be the long sought-after NMDAR antagonist for potent and specific inhibition of morphine dependence with minimal side effects, offering great potential for clinical applications.

## 2. Results

### 2.1. Con-T[M8Q] Potently Inhibits the Expression and Development of Morphine Physical Dependence in Mice

**Figure 1:**
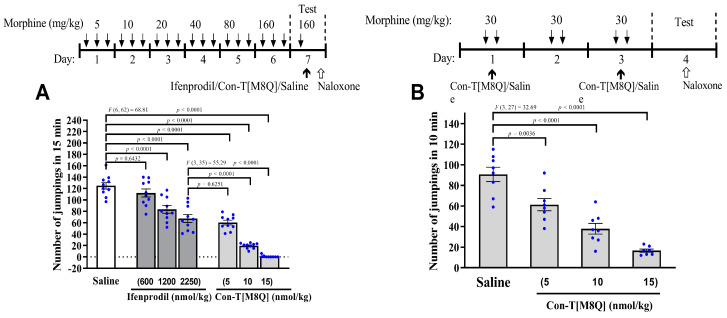
Con-T[M8Q] potently inhibits the expression and development of physical dependence to morphine in mice. (**A**) The effects on the expression of morphine-induced physical dependence of con-T[M8Q](*n* = 8–10); (**B**) The effects on the development of morphine-induced physical dependence of con-T[M8Q] (*n* = 8). The upper of figures is the schematics of the morphine administration/behavioural paradigm. All morphine-dependent mice received naloxone (i.p.) at 4.5 mg/kg to induce jumping. The number of jumps over a 15-min (**A**) or 10-min (**B**) period is expressed as mean ± SEM. The control groups without morphine injection shows no jumping after the administration of naloxone. Data analysed by separate one-way analysis of variance (ANOVA) followed by the Dunnett’s multiple comparison test. (**A**): F (6, 62) = 68.81, *p* < 0.0001 ifenprodil group (1200, 2250 nmol/kg) and con-T[M8Q] vs. saline control; F (3, 35) = 55.29, *p* < 0.0001 con-T[M8Q] (10, 15 nmol/kg) vs. ifenprodil group (2250 nmol/kg). (**B**): F (3, 27) = 32.69, *p* < 0.005 or *p* < 0.0001 con-T[M8Q] vs. saline group.

As shown in Figure 1A, con-T[M8Q] (intracerebroventicular injection, i.c.v.) significantly inhibits naloxone-induced jumping in morphine-dependent mice at 5, 10, and 15 nmol/kg compared to saline groups (Figure 1A) and ifenprodil. At 15 nmol/kg, con-T[M8Q] almost completely inhibits naloxone-induced jumping, which is 100-fold more potent compared to ifenprodil (2250 nmol/kg) using the same administration route (*p* < 0.0001). Con-T[M8Q] (i.c.v.) significantly inhibits naloxone-induced jumping in a dose-dependent manner. Similar results were observed for the inhibition of the development of morphine physical dependence in mice (Figure 1B).

### 2.2. Con-T[M8Q] Potently Inhibits the Expression and Development of Morphine-Induced CPP in Mice

**Figure 2:**
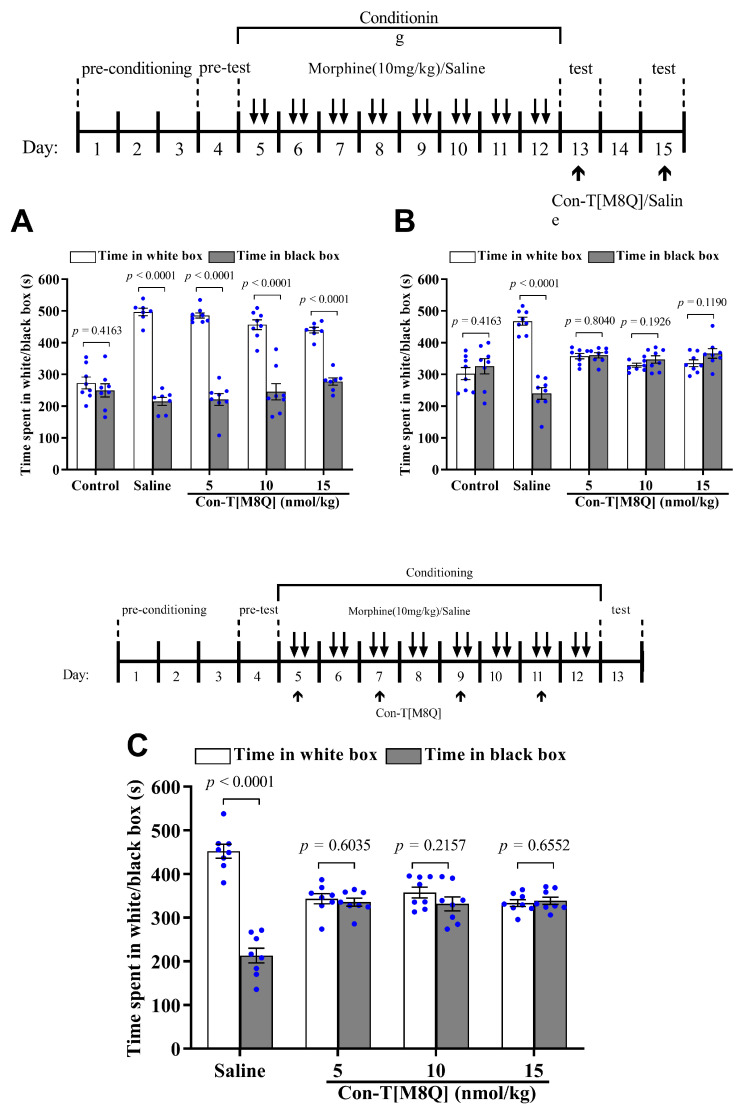
Con-T[M8Q] efficiently inhibits the expression and development of morphine-induced CPP in mice (*n* = 8). (**A**) The effects on the expression of morphine-induced CPP after the administration of con-T[M8Q] on day 13; (**B**) The effects on the expression of morphine-induced CPP after administration of con-T[M8Q] on day 15; (**C**) The effects on the development of morphine-induced CPP on day 13 after the administration of con-T[M8Q] on days 4, 7, 9, and 11. The upper of figures is the schematics of the morphine administration/behavioural paradigm. The time spent in the white (drug side) or black (saline side) chamber was recorded and was expressed as mean ± SEM. The data were analysed by unpaired t-test. (**A**), morphine dependent mice, *p* < 0.0001 white chamber vs. black chamber. (**B**), *p* < 0.0001 white chamber vs. black chamber (morphine dependent mice with saline), *p* > 0.05 white chamber vs. black chamber (other groups). (**C**), *p* > 0.05 white chamber vs. black chamber (con-T[M8Q] groups).

Next, we wanted to test for the inhibitory effects of con-T[M8Q] on psychological morphine dependence. In a conditioned place preference (CPP) experiment, eight days after the administration of morphine, mice stayed for longer time in the white (initially non-preferred) chamber and successfully demonstrated morphine-induced CPP. After the first administration (i.c.v.) of con-T[M8Q] (5 or 10 or 15 nmol/kg) on day 13, the mice still spent a significantly higher amount of time during the 15-min session in the white (drug) versus black (saline) side of the chamber. This suggests that one injection of con-T[M8Q] is not enough to inhibit the morphine-induced CPP. Therefore, we gave the mice the same dose of con-T[M8Q] (5, 10, and 15 nmol/kg, i.c.v.) or saline again on day 15. This time, the results show that the morphine-induced CPP in mice was efficiently inhibited (Figure 2B), with almost identical (*p* > 0.05) time spent between the white and black chamber in the con-T[M8Q] groups. In contrast, in the saline group, mice continued to show a preference for staying in the white chamber. Similar results were found in the development of CPP (Figure 2C). After the administration of morphine for 8 days (on days 4–12) and con-T[M8Q] (5, 10, and 15 nmol/kg) on days 5, 7, 9, and 11, the time spent in the white versus black chamber was not significantly different in con-T[M8Q]-treated groups on day 13. However, in the saline group (i.c.v.), mice showed apparent CPP (Figure 2C). These results indicate that con-T[M8Q] potently inhibits the development of morphine-induced CPP in mice.

### 2.3. Con-T[M8Q] Has Little Effects on the Coordinated Locomotion and Spontaneous Locomotor Activity in Mice

**Figure 3:**
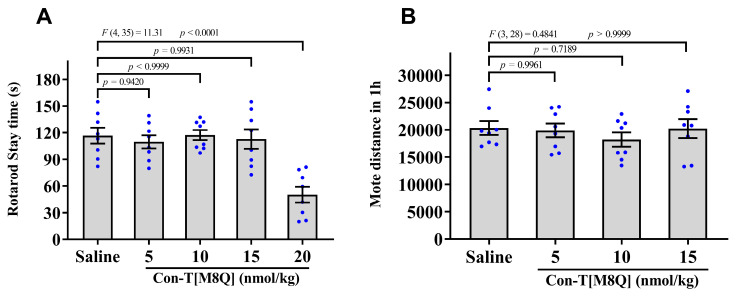
Con-T[M8Q] has little effects on motor function and spontaneous locomotor activity (SLA) in mice. (**A**) The stay times spent on the rod after administration of saline and con-T[M8Q]; (**B**) Distance of SLA of mice 1 h after administration of con-T[M8Q]. Mice (*n* = 8) were injected with con-T[M8Q] (i.c.v.) at 0, 5, 10, and 15 nmol/kg in both experiments. Data represent the mean ± SEM. The data were analysed by separate one-way analysis of variance (ANOVA) followed by the Dunnett’s multiple comparison test. (**A**), F (4, 35) = 11.31, *p <* 0.0001 con-T[M8Q] (20 nmol/kg) vs. saline. (**B**), F (3, 28) = 0.4841, *p* > 0.05 con-T[M8Q] (5,10, 15 nmol/kg) vs. saline.

The typical side-effects of conantokins are motor disorders and abnormalities in the nervous system, so we investigated the effects of con-T[M8Q] on coordinated locomotion function and spontaneous locomotor activity of mice. After the administration of con-T[M8Q] (5, 10, and 15 nmol/kg), the time spent on the rod was not significantly different and was almost identical to that for the saline group (Figure 3A). However, a high dose of con-T[M8Q] (20 nmol/kg) produced the inhibitory activity in the motor function, and the time spent on the rod decreased, so higher doses were not used. Considering the fact that this dose is already beyond what is needed for full inhibition of morphine dependence in naloxone-induced jumping and CPP experiments, the side effects of con-T[M8Q] are quite low. Similarly, no significantly inhibitory activity was observed in the spontaneous locomotor activity of mice after the administration of con-T[M8Q] (5, 10, and 15 nmol/kg) in 1 h (Figure 3B), 2 h and 3 h (data not shown).

### 2.4. Con-T[M8Q] Has No Apparent Effects on the Spatial learning Memory and Menory Recall in Mice

**Figure 4:**
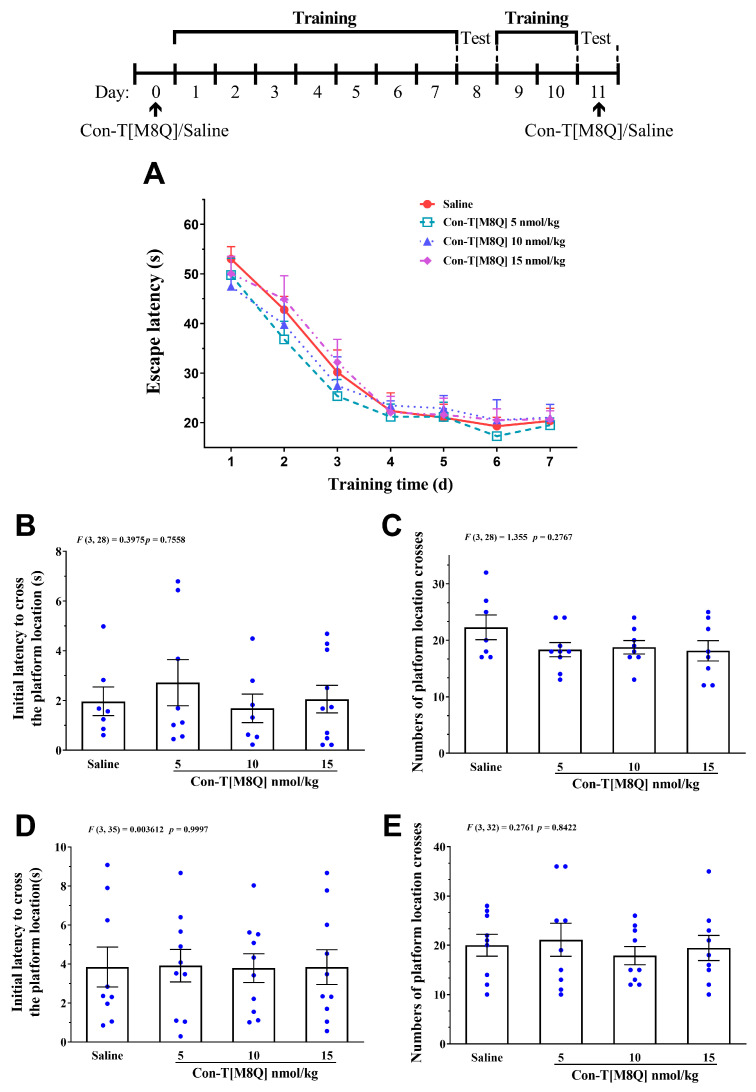
Con-T[M8Q] has no apparent effect on learning and memory in mice. (**A**) Escape latency to reach the platform on day 1–7, mice (*n* = 10–12) received saline (i.c.v.) and con-T[M8Q](5, 10,15 nmol/kg, i.c.v.) on day 0; (**B**) Initial latency (time) to cross the platform location in probe trials on day 8; (**C**) Numbers of platform location crosses in the probe trial on day 8; (**D**) Initial latency (time) to cross the platform location in probe trials on day 11; (**E**) Numbers of platform location crosses in the probe trial on day 11, the data (**D**,**E**) were collected 1 h after administration of saline (i.c.v.) and con-T[M8Q](5, 10, 15 nmol/kg, i.c.v.) on day 11. Data are represented as the mean ± SEM, *p* > 0.05 in all groups.

After con-T[M8Q] (5, 10, and 15 nmol/kg, i.c.v.) was administrated at the begin of training trial, no significant effects were observed on the escape latency of mice in 7 consecutive days (Figure 4A) (*p* > 0.05) and the initial latency to reach the platform location (Figure 4B) (*p* > 0.05) and number of platform location crosses (Figure 4C) (*p* > 0.05) on day 8. These results suggest that con-T[M8Q] does not affect spatial learning memory. In order to investigate the effects of con-T[M8Q] on the existing spatial memory, mice were sequentially trained for two days and the probe trials were determined again on day 11 after second administration of con-T[M8Q] (5, 10, 15 nmol/kg), the results showed that con-T[M8Q] displayed similar performances compared to the blank control mice (Figure 4D,E) (*p* > 0.05), suggesting that con-T[M8Q] does not affect the spatial memory recall in mice.

### 2.5. Con-T[M8Q] Decreases the Levels of GluN2B, CaMKs, nNOS and c-fos mRNA in Morphine-Dependent Mice

**Figure 5:**
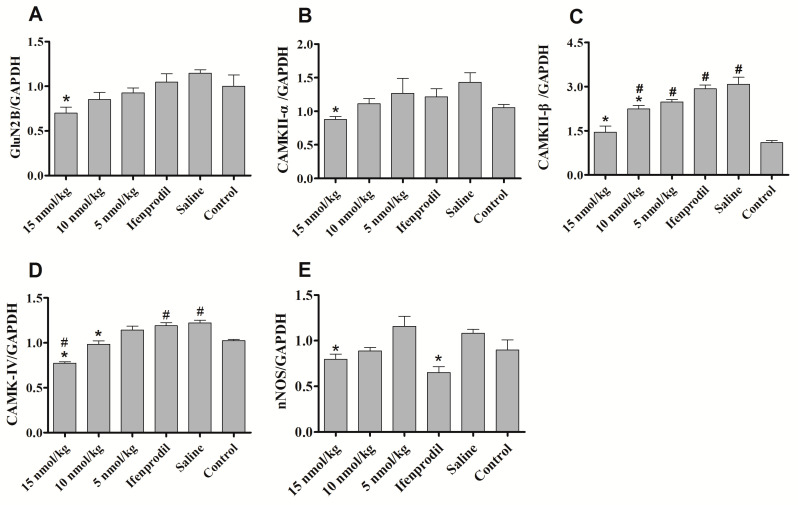
Effects of con-T[M8Q] on the expression of NMDAR-related signalling molecules of morphine dependent mice at the transcript level. The mRNA levels of GluN2B, CaMKII-α, CaMKII-β, CaMKIV, nNOS in blank control mice treated with saline (N) and morphine-dependent mice treated with saline, con-T[M8Q] (5 nmol/kg, 10 nmol/kg and 15 nmol/kg) and Ifenprodil (1250 nmol/kg). (**A**) GluN2B; (**B**) CaMKII-α; (**C**) CaMKII-β; (**D**) CaMKIV; (**E**) nNOS. All samples were analyzed in three independent experiments. Each bar is the mean ± SEM of independent determinations in 8 mice per group. Relative mRNA expression = ratio of specific mRNA over GAPDH mRNA. * *p* < 0.05 vs. saline group, ^#^ *p* < 0.05 vs. blank control (morphine-independent mice).

There is increasing evidence suggesting that NMDAR in hippocampus, striatum, ventral tegmental area and nucleus accumbens is involved in morphine/opiate dependence [23,24,25,26,27]. It has been reported that the levels of NR2B were elevated in the hippocampus and nucleus accumbens in the morphine-dependent mice [8,23]. Hence, we also analyzed the immunofluorescence changes of GluN2B in hippocampus and whole brain of morphine-dependent mice. The results showed that the immunofluorescence of GluN2B increased mainly in hippocampus of morphine-dependent mice, but the expression of NR2B decreased significantly after treatment of con-T[M8Q] (data not shown). Based on the preliminary results of GluN2B immunofluorescence, we then examined the effects of con-T[M8Q] on the mRNA levels of the NMDAR-related signaling molecules such as GluN2B, CaMK gene members, nNOS and PKC-γ in the hippocampus. The results show that 10 or 15 nmol/kg of con-T[M8Q] significantly inhibits the mRNA expression level for GluN2B in the hippocampus (Figure 5A). The same results are observed for CaMK proteins (including CaMKII α, β and CaMKIV) (Figure 5B–D) and nNOS (Figure 5E). However, con-T[M8Q] has no apparent effects on the mRNA expression of PKC-γ (data not shown).

### 2.6. Con-T[M8Q] Efficiently Decreases the Expressions of GluN2B, p-GluN2B, CaMKs, nNOS, pERK and c-fos Proteins in Morphine-Dependent Mice

**Figure 6:**
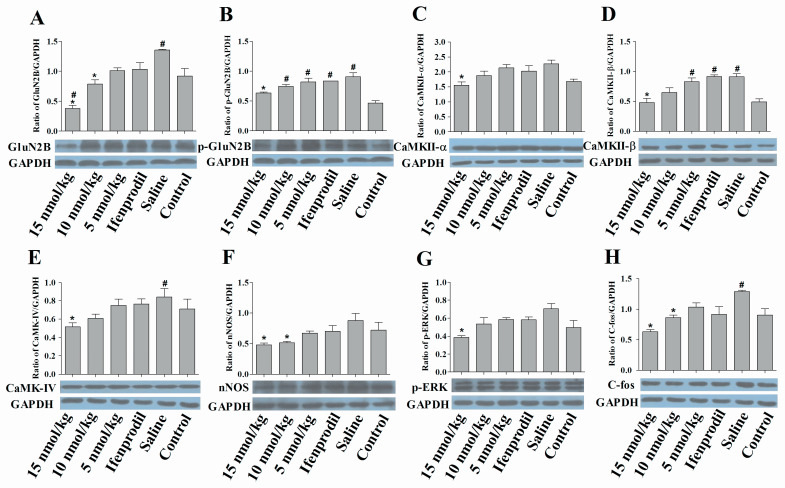
Con-T[M8Q] efficiently decreases the expression of NMDAR-related signaling molecules of morphine dependent mice at the protein level. Western blots of NMDAR-related signal molecules in hippocampal extracts from the morphine-dependent mice treated with saline, con-T[M8Q] and ifenprodil (1250 nmol/kg), and blank control mice treated with saline. GAPDH was the control in all experiments. GluN2B (**A**); p-GluN2B (**B**); CaMKII-α (**C**); CaMKII-β (**D**); CaMKIV (**E**); nNOS (**F**); p-ERK (**G**); c-fos (**H**). The proteins were immunostained with the corresponding antibody and an anti-rabbit IgG secondary antibody conjugated to HRP (1:10000). Each bar is the mean ± SEM of independent determinations in 6 mice per group. * *p* < 0.05 vs. saline group, ^#^ *p* < 0.05 vs. blank control (morphine-independent mice).

As shown in Figure 6, the expression levels of GluN2B, p-GluN2B and CaMKII-β proteins in morphine-dependent mice are higher than the blank control mice. 15 nmol/kg of con-T[M8Q] significantly reduced the expressions of GluN2B protein in the hippocampus of morphine-dependent mice in a dose-dependent manner (Figure 6A) as expected from the above mRNA analysis. It has been reported that phosphorylated GluN2B at Tyr1472 is involved in the neuropathic pain [28,29,30], so the effects of con-T[M8Q] on the phosphorylation of Tyr1472 of GluN2B were examined. The results show that the phosphorylation of Tyr1472 of GluN2B is inhibited by the higher dose of con-T[M8Q] (15 nmol/kg, Figure 6B). Similar results were found in CaMKII-α, β (Figure 6C,D), CaMKIV (Figure 6E) and pERK proteins (Figure 6G). Con-T[M8Q] (15 nmol/kg) also displays significant inhibitory activities to the expressions of nNOS (Figure 6F) and c-fos (Figure 6H).

## 3. Discussion

In the previous study, we found that that con-T and other variants (the non-selective inhibitors for NMDAR GluN2B [31]), such as con-T[M8A], con-T[M8F], con-T[M8I] and con-T[M8N], could inhibit the expression and development of morphine physical dependence. In fact, Con-T[M8Q] was also tested along with the above con-T and its variant though the data of con-T[M8Q] were not reported. The results showed that con-T[M8Q] more efficiently inhibits the expression and development of morphine physical dependence than that of con-T and other variants. For example, at same dose of 15 nmol/kg, con-T[M8Q] almost completely inhibits naloxone-induced jumping in the acute expression experiment model but not for con-T and other variants (40~80 jumping numbers) [31]. In the development of morphine physical dependence, con-T[M8Q] shows higher or same inhibitory activity compared to con-T and other variants. Therefore, these results led to the further investigation of con-T[M8Q] on morphine dependence.

Con-T[M8Q] also efficiently inhibits the expression and development of morphine psychological dependence in mice at the nmol/kg level (Figure 2A) after repeated administration. To the best of our knowledge, con-T[M8Q] is the first GluN2B peptide inhibitor that has been reported to potently inhibit morphine psychological dependence. Con-T[M8Q] displays significantly higher inhibitory activity and lower side effects for mouse motor function and spontaneous locomotor activity (Figure 3). It also has not apparent effects on mice spatial learning memory and memory recall (Figure 4). In addition, we found that con-T[M8Q] did not bind to opiate receptors (Supplementary Table S1) and the voltage-gated potassium, sodium channels in DRG cells at 10 μM (inhibition ratio was <5%, data not shown). These results suggest that the high inhibitory activity of con-T[M8Q] for morphine dependence and lower side effects are mainly attributed to its selective inhibition of the NMDAR GluN2B subunit. Of course, non-selective NMDAR GluN2B inhibitors conantokins also inhibit morphine dependence.

A number of signalling proteins have been found to be involved in morphine dependence [9,32,33,34,35]. NMDAR/nNOS/CaMKII signal pathway plays a critical role in the cross-regulation of Mu-opioid and NMDAR [9]. To probe the mechanism of how con-T[M8Q] inhibits morphine dependence with such a high potency, the effects of con-T[M8Q] on the expression of NMDAR-related signal proteins, such as GluN2B, p-GluN2B, CaMKII-α, CaMKII-β, CaMKIV, nNOS, PKC-γ, and c-fos, were investigated. Before the administration of con-T[M8Q], the expression of GluN2B, p-GluN2B,c-fos and CaMKII-β proteins increases significantly after morphine administration, consistent with the literature [36,37,38,39,40]. After the administration of con-T[M8Q], the increased mRNA or protein levels of above proteins decrease significantly in the hippocampus (Figure 5 and Figure 6). The decreases in the expressions of GluN2B and p-GluN2B may be derived from the cross-regulation of Mu-Opioid [9] as well as the decreases of calcium flow, and the latter further reduces the deactivation of CaMKII [22,37]. In addition, we found that con-T[M8Q] does not induce morphine-like dependence [22] and the binding of con-T[M8Q] (10 μM) to opioid receptor is very low (Supplementary Table S1). These results suggest that the NMDAR/nNOS/CaMKII may be the key signaling pathway for con-T[M8Q] inhibition of morphine dependence. It is known that cAMP response-element binding protein (CREB), a transcription factor involved in learning, memory and drug addiction, is phosphorylated by CaMKIV [41], and CaMKIV-knockout mice have less analgesic tolerance to morphine [42,43]. Our results show that the expression of CaMKIV increases in morphine-dependent mice and this increase is efficiently inhibited by con-T[M8Q], suggesting that CaMKIV may also be regulated by NMDAR. However, ifenprodil displays lower effects on the above proteins than that of con-T[M8Q] in the hippocampus of physical morphine-dependent mice. The more detailed mechanism of morphine dependence regulated by NMDAR is highly complex. We believe con-T[M8Q] provides a useful molecular tool to dissect this complex network in the future.

Previous experiments demonstrate that ifenprodil bind to the N-terminal of GluN2B [44,45,46]. Conantokins act at allosteric modulatory sites associated with polyamine sites [47]. Con-G, another GluN2B subunit-selective inhibitor, binds to the S2 regions of the agonist-binding domain of the GluN2B subunit [48]. Thus, the binding site of con-T[M8Q] in GluN2B subunit may also be different from ifenprodil, and the allosteric modulation of con-T[M8Q] may be more efficiently [49] because conantokins display similar inhibitory activities in the radioligand binding experiments of rat or human brain membranes [50,51]. The target specificity and moderate binding ability of con-T[M8Q] may be the reason for its low side-effects.

In conclusion, we found that the NMDAR GluN2B specific antagonist con-T[M8Q] exhibit highly potent inhibitory activity effects on physical and psychological morphine dependence at the nmol/kg level, significantly more potent than ifenprodil, a typical GluN2B specific inhibitor. Importantly, con-T[M8Q] does not have significant side-effects on motor function. The high potency and specificity of con-T[M8Q] may be derived from the effective inhibition of the expression of GluN2B and subsequent down-regulation of CaMKII, CaMKIV, nNOS, pERK and c-fos signalling proteins. Therefore, we believe that con-T[M8Q] is a candidate for the clinical treatment of morphine dependence.

## 4. Materials and Methods

### 4.1. Animals and Drugs

Kunming mice (18–22 g, 3–4-week old) were housed in groups of eight on a 12-h light–dark cycle (light cycle from 8 am to 8 pm) at 23 ± 2 °C and a relative humidity of 50%. Food pellets and water were available *ad libitum*. All experiments were conducted in accordance with the guidelines of the Animal Research Advisory Committee of the Beijing Institute for Biological Sciences (Beijing, China) and conformed to European Community directives for the care and use of laboratory animals.

Con-T[M8Q] (GEγγYQKMLγNLRγAEVKKNA-NH_2_) was synthesized and purified as previously described [52,53]. Morphine sulphate and naloxone were purchased from Northeast Pharmaceutical Group Co. LTD. (Shenyang, China) and Sihuan Pharmaceutical Co., LTD (Beijing, China), respectively. Anti-GluN2B and anti-phospho-GluN2B antibodies were supplied by Sigma-Aldrich (St Louis, MO, USA). Antibodies for CaMKII-α, CaMKII-β, and phospho-ERK and horseradish peroxidase (HRP)-labelled secondary antibody were obtained from CST Corporation (Cell signaling Technology, Danvers, MA, USA). The antibodies for nNOS, CaMKIV, and c-fos were purchased from Abcam Corporation (Cambrige, UK). A real-time reverse-transcription-polymerase chain reaction (RT-PCR) assay kit was purchased from Promega (Madison, WI, USA).

### 4.2. Morphine Physical Dependent Abstinence/Withdrawal

For the inhibition of expression of morphine dependence, male kunming mice (20 ± 2 g) were randomly divided into the morphine dependence groups and the control group without morphine administration (8–10 animals in each group), the morphine dependence groups were treated with antagonist groups or saline. Mice were injected subcutaneously (s.c.) three times daily (t.i.d.: 8:00, 16:00 and 20:00) for 7 days according to an escalating dose schedule [18,54,55]. The initial dose of morphine was 5 mg/kg. Thereafter, the dose of morphine was doubled every second day. A dose of 160 mg/kg was delivered on the sixth day. On the morning of the seventh day, 10 μL of saline (0.5 mL/kg), con-T[M8Q] (5, 10 and 15 nmol/kg, dissolved in saline) or ifenprodil (600, 1200 and 2250 nmol/kg, dissolved in saline) was administrated (i.c.v) to mice 2.5 h following the final dose of morphine (160 mg/kg). The abstinence syndrome was precipitated by administering an i.p. injection of naloxone (4.5 mg/kg, 400 μL) 30 min after injection of saline or the NMDAR antagonists. Each mouse was immediately placed in a square observation box (30 cm × 30 cm × 50 cm) and the number of jumps was recorded over a 15 min period.

To avoid the possible effects of injection (i.c.v.) times on mice brain, the three-day model was used in the inhibition of the development of morphine dependence according to the reported method [56] and made some modifications. This model induces moderate morphine physical dependence, and the shorter experimental time decreases times of injection (i.c.v.). Briefly, mice received morphine (30 mg/kg, s.c.) at 8 am and 4 pm at days 1–3. On day 1 and day 3 morning, mice received 10 μL of con-T[M8Q] (5, 10, and 15 nmol/kg) or saline 30 min after morphine treatment. All mice were challenged with 4 mg/kg naloxone on the morning of the 4th day, and the number of withdrawal jumps in 10 min was immediately recorded.

### 4.3. Morphine Psychological Dependent Abstinence

The morphine psychological dependence was assessed by CPP in male mice as described previously [18,55]. The CPP procedure consisted of three phases, such as pre-conditioning phase (4 days), conditioning training (8 days) and test phase (1–3 days). The apparatus consists of sixteen identical plastic boxes with white chambers (dimension 30 × 20 × 25 cm, plain wood floor), black chambers (dimension 30 × 20 × 25 cm, wire mesh floor) and a grey central tunnel (12 × 20 × 25 cm). Each central tunnel was connected to the corresponding white chamber and black chamber via a guillotine baffle. The time spent of mice in black and white chamber was recorded by a camera on the top. During the first 3 days of training (pre-conditioning phase), mice were placed individually in the central tunnel and freely explored three compartments for 10 min every day. On day 4 (pre-test), the time spent in the white chamber during a 15-min free exploration was measured and recorded. This measurement was used as an initial preference score for each mouse. 10 of 64 mice was excluded because of a strong preference for one chamber, which they spent more than 200 s in a chamber than in another chamber. Then, the mice used in this experiment did not show typical a clear preference.

To investigate the effects of con-T[M8Q] on the expression of morphine-induced CPP, the qualified mice were randomly divided into five groups (*n* = 8). On days 5, 7, 9, and 11, mice of each group were injected with saline (s.c.) at 8.30 am or morphine (10 mg/kg, s.c.) at 4 pm. On days 6, 8, 10, and 12, mice were injected with morphine (10 mg/kg, s.c.) at 8.30 am and saline at 4 pm. After the administration of morphine or saline, mice were put in morphine-paried white chamber or saline-paired black chamber for 45 min, respectively. The conditioning trials lasted for 8 days. 30 min after the administration of saline (10 μL, i.c.v.) or con-T[M8Q] (5, 10, and 15 nmol/kg, i.c.v., 10 μL) on day 13 and 15, mice were placed in the central tunnel and the time spent in the white or black chamber in 15 min was recorded.

To investigate the effects of con-T[M8Q] on the development of CPP, the experimental procedure, dose and time of administration of morphine in the formation of CPP mice were the same as the expression of CPP in mice mentioned above. Briefly, con-T[M8Q] (5, 10, and 15 nmol/kg, 10 μL) or saline (10 μL) was injected into mice (i.c.v.) on days 5, 7, 9, and 11 for each group after the administration of morphine or saline. Mice were then placed in the central tunnel. The time spent in the white or black chamber on day 13 in 15 min was recorded.

### 4.4. Coordinated Locomotion in Rotarod Test

Motor impairment was determined on an accelerating rotarod treadmill (Ugo Basile, Comerio, Italy) as previously described [19,57]. Briefly, con-T[M8Q] (5, 10, 15, 20 nmol/kg), or saline was administered i.c.v. to the male mice (*n* = 8) in a volume of 10 μL. At 60 min following injection, the mice were placed on the rotating rod at a speed of 4 rpm. After 50 s, the rod was accelerated from 4 rpm to 40 rpm over a 5 min interval. The time that the mice remained balanced on the rod was recorded.

### 4.5. Spontaneous Locomotor Activity

Kunming mice were randomly divided into several dose groups and a saline (icv) group with 8 animals in each group, half females and half males. Horizontal locomotor activity was recorded in boxes (20 × 31 × 13 cm) fitted in frames equipped with infrared beam systems (NS-AS01; Neuroscience Inc., Tokyo, Japan). 30 min after the administration (i.c.v.) of con-T[M8Q] (5, 10, 15 nmol/kg) or saline, the spontaneously moving distance was automatically recorded for 3 h.

### 4.6. Morris Water Maze Test

The Morris water maze test was performed to assess the effects of con-T[M8Q] on mice spatial learning and memory as described previously [58]. Briefly, kunming mice (half male and half female, 22 ± 2 g) were randomly divided into several dose groups and a saline control group with 10–12 animals in each group. In order determine to the effects of con-T[M8Q] on the memory formation, one day before training trial, mice received saline (i.c.v.) and con-T[M8Q](5, 10, 15 nmol/kg, i.c.v.), and were then trained to find the invisible submerged black platform (1 cm below the water surface) in a circular pool (1.0 m in diameter and 0.5 m high) (DMS-2 Morris water maze test system, Institute of Materia Medica, Chinese Academy of Medical Sciences) filled with water (25 ± 1 °C) using a variety of visual cues located on the pool wall. Four trials were performed each day for 7 consecutive days. On each trial, the mouse was released into the pool at one of four designated start locations and allowed to find and climb onto the hidden platform. If a mouse failed to find the platform with in 60 s, it was manually guided to the platform and allowed to remain there for 20 s. The escape latency was recorded as the time from being placed into the water to climbing the escape platform during training. On day 8, after the training trials, a probe trial was performed by removing the platform. Mice were allowed to swim for 60 s in search of the platform and were monitored by a video camera and analyzed by a computer. In order to further determine the effects of con-T[M8Q] on the existing spatial memory, mice were sequentially trained for two days after probe trial. On day 11, 1 h after the administration of saline (i.c.v.) and con-T[M8Q] (5, 10, 15 nmol/kg, i.c.v.), the probe trial was performed again to assess the effects of con-T[M8Q] on mice spatial memory. The parameters measured during the probe trial were the initial latency to reach the platform location and number of platform location crosses.

### 4.7. mRNA Extraction and Quantitative RT-PCR

The morphine dependent models were constructed as described in the above experiment of expression of morphine physical dependence. Mice (8 animals in each group) were then injected (i.c.v.) with saline, con-T [M8Q] (5, 10 and 15 nmol/kg) and ifenprodil (1250 nmol/kg) at 8.30 am for three days. 2.5 h after the last administration, the mice were killed, and the hippocampus of each group was dissected, mixed and immediately frozen in liquid nitrogen. Total RNA was extracted and purified from 100 mg of homogenised hippocampus using Trizol reagent (Invitrogen, Carlsbad, CA, USA). One microgram of total RNA from each group was reverse-transcribed into cDNA using reverse transcriptase M-MLV with oligo-dT18. Each synthesised cDNA was used as the template for qRT-PCR reactions. The reaction system included forward and reverse primers (Table S2), 2x SYBR Premix Ex Taq TM II (supplied in the kit) and a template. qRT-PCR was carried out as follows: 95 °C for 10 min; 95 °C for 10 s, different annealing temperature (Table S2) for 15 s, and 72 °C for 12 s (40 cycles). PCR product was measured using SYBR Green fluorescence collected on a Biorad IQ5 system. Melting point analyses were performed for each reaction to confirm single amplified products. 2^-ΔΔCt^ analysis was used to normalize the mRNA level to that of GAPDH. Data are presented as relative mRNA expression compared to control values. The Ct value of experiment is controlled from 18 to 25.

### 4.8. Western Blot Analyses

The morphine dependent mice, con-T[M8Q] administration procedures and brain tissue separation were the same as “mRNA extraction and quantitative RT-PCR” section. After extraction of total protein from the forebrain or hippocampus of eight mice in each group, protein was quantified using a bicinchoninic acid (BCA) assay kit (Pierce, Rockford, IL, USA) and 20 μg of proteins were used for sodium dodecyl sulfate-polyacrylamide gel electrophoresis (SDS-PAGE). After electrophoretic transfer and blocking with 5% non-fat milk, membranes were incubated with the primary antibody [anti-CaMKIIa/β (dilution, 1:2000), anti-CaMKIV (1:5000), anti-GluN2B (1:1000), anti-phosphorylated GluN2B (1:1000), anti-c-fos (1:1000), anti-nNOS (1:1000), anti-phosphorylated ERK (1:2000), anti-GAPDH (1:2000)] in TBS/T buffer at 4 °C overnight. Membranes were then incubated with anti-rabbit IgG secondary antibody conjugated to HRP (1:5000). The complexes on the membrane were visualised using an enhanced chemiluminescence (ECL) detection system (Pierce). GAPDH was used as an internal control. Image-Pro plus software was used to measure the mean optical densities (OD) and areas of protein signal on radiographic film after scanning. The data were presented as relative protein expression compared to that of GAPDH protein.

### 4.9. Statistical Analyses

The data of physical morphine dependences, Morris water maze tests, qRT-PCR experiments and Western blot experiments were analysed by separate one-way analysis of variance (ANOVA) followed by the Dunnett’s multiple comparison test at the 0.05 level of significance. The data of psychological morphine dependences were analysed by unpaired t-test. The data of animal experiments and the biochemical data were expressed as mean ± SEM.

## Data Availability

Data is contained within the article or supplementary material.

## Supplementary Materials

The following are available online at https://www.mdpi.com/1660-3397/19/1/44/s1, Table S1: The binding ability of con-T[M8Q] to opioid receptor; Table S2: The primers and sequences used for qRT-PCR.
