# Supplementary material for: A Conantokin Peptide Con-T[M8Q] Inhibits Morphine Dependence with High Potency and Low Side Effects
Source: Mar Drugs. 2021 Jan 19;19(1):44. doi: 10.3390/md19010044 (PMC7835912; doi:10.3390/md19010044)
Supplement: Supplementary file 1 [file marinedrugs-19-00044-s001.pdf]

A Conantokin Peptide Con-T[M8Q] Inhibits Morphine Dependence with High Potency and Low Side Effects

Zhuguo Liu[1,†], Zheng Yu[1,†], Shuo Yu[1, †], Cui Zhu[1,], Mingxin Dong[1], Wenxiang Mao[1], Jie Hu[1], Mary Prorok[3], Ruibin Su[2,*] and Qiuyun Dai[1,*]

[1] Beijing Institute of Biotechnology, Beijing 100071, China; liuzhuguo@126.com (Z.L.); YZYZ.6688@163.com (S.Y.); o-yys@163.com (S.Y.); zhucuililac@126.com (C.Z.); mxdong64@aliyun.com (M.D.); maomao@sina.cn (W.M.); hujie0906@126.com (J.H.); daiqy@bmi.ac.cn (Q.D.)

[2] Department of Chemistry and Biochemistry, University of Notre Dame, Notre Dame, Indiana 46556, USA; mprorok@nd.edu

[3] Beijing Institute of Toxicology and Pharmacology, Beijing 100850, China; surb@bmi.ac.cn

Opioid receptor binding assay

Ligand binding experiments were carried out with [^{3}H]diprenorphine for opioid receptors as described previously (1,2). Competition inhibition by peptides and morphine of [^{3}H]diprenorphine binding to opioid receptors was performed in the absence or presence of various concentrations of each drug. Binding was carried out in 50mM Tris.HCl buffer (pH7.4) at 37℃ for 30min in triple in a final volume of 0.5ml with 30 μg of membrane protein prepared from CHO cell expressing human κ-, rat μ- or rat δ- opioid receptors. Naloxone (10μM) was used to define nonspecific

binding. Bound and free [^{3}H]diprenorphine were separated by filtration under reduced pressure with GF/B filters. Radioactivity on filters was determined by liquid scintillation counting.

Table S1. The binding ability of con-T[M8Q] to opioid receptor

Opioid receptor	μ-receptor	K-receptor	δ-receptor
Con-T[M8Q] (μM)	5	5	5
Inhibition (%)	13.1 ±0.1	7.1 ±0.8	0

Table S2. The primers and sequences used for qRT-PCR

Gene	Primer sequences	Annealing temperature℃
**GAPDH**	F: CAA GGC TGA GAA TGG GAA G	58
	R: TGG TGA AGA CGC CAG TAG A	58
**GluN2B**	F: ACT AAC TAT CAA TGA AGA ACG GT	60
	R: AGG AGA GGA AGA GCT ACA A	58
**CAMKII-α**	F: ATC GCC TAT ATC CGC ATC AC	60
	R: GGA CAA AGA GCG GAT CTC TG	60
**CAMKII-β**	F: CAT TGT ACG CCT CCA TGA CA	58
	R: GGA TGC AGT GAC TGG CAT CA	58
**CaMK-IV**	F: CGG CTG ACT ACA TTT CAA GCC	56
	R: CTT CAC CGC TGC CTT AAG CTT	58
**nNOS**	F: AGG AGA GGA AGA GCT ACA A	56
	R: AAG ACT GAG AAC CTC ACA TT	56
**PKC-γ**	F: CAA CTT CAT TCC ACC TTT CAG A	50
	R: GCA TCC AGC ATC ACA TTA TCC	50

References

1. Li, W., Tao, Y.M., Tang, Y., Xu, X.J., Chen, J., Fu, W., Wang, X.H., Chao, B., Sheng, W., Xie, Q., Qiu, Z.B., Liu, J.G. Highly selective and potent mu opioid

ligands by unexpectedsubstituent on morphine skeleton. *Bioorg. Med. Chem. Lett.* **2010**, *20*, 418-421.

2. Liu, J.G., Prather, P.L. Chronic exposure to mu-opioid agonists produces constitutive activation of mu-opioid receptors in direct proportion to the efficacy of the agonist used for pretreatment. *Mol. Pharmacol*. **2001**, *60*, 53-62.
